# Synergistic Effect of Binary Surfactant Mixtures in Two-Phase and Three-Phase Systems

**DOI:** 10.1021/acs.jpcb.1c00664

**Published:** 2021-04-13

**Authors:** Agata Wiertel-Pochopien, Piotr Batys, Jan Zawala, Przemyslaw B. Kowalczuk

**Affiliations:** † Jerzy Haber Institute of Catalysis and Surface Chemistry, Polish Academy of Sciences, Niezapominajek 8, 30-239 Krakow, Poland; ‡ Department of Geoscience and Petroleum, Norwegian University of Science and Technology, S. P. Andersens veg 15a, 7031 Trondheim, Norway; § Faculty of Chemistry, Wroclaw University of Science and Technology, Wybrzeze Wyspianskiego 27, 50-370 Wroclaw, Poland

## Abstract

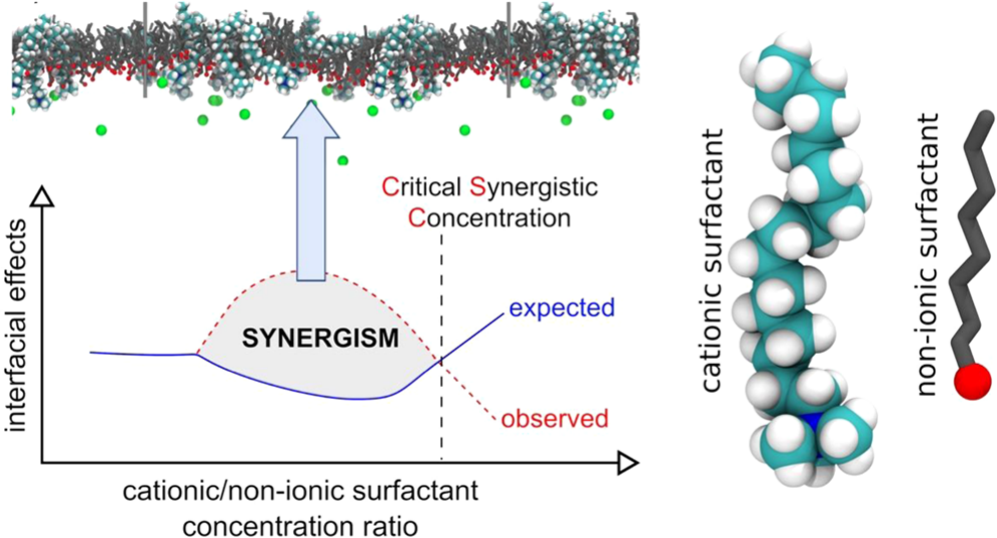

Cationic alkyltrimethylammonium bromides (C*_n_*TAB, with *n* = 8, 12, 16, 18) and their mixtures with *n*-octanol as a nonionic surfactant were chosen as a model system to study the synergistic effect on foamability (two-phase system) and floatability (three-phase system) of quartz in the presence of binary mixtures of ionic/nonionic surfactants. The foam height of one-component solutions and binary mixtures and floatability of quartz particles were characterized as a function of the surfactant concentration and the number of carbons (*n*) in the alkyl chain of C*_n_*TAB. The experimental results of foamability and floatability measurements in one-component and mixed solutions revealed the synergistic effect, causing a significant enhancement in the foam height and recovery of quartz. In the presence of *n*-octanol, the height of foam increased remarkably for all C*_n_*TAB solutions studied, and this effect, whose magnitude depended on the C*_n_*TAB hydrophobic tail length, could not be justified by a simple increase in total surfactant concentration. A similar picture was obtained in the case of flotation response. The mechanism of synergistic effect observed in mixed C*_n_*TAB/*n*-octanol solutions was proposed. The discussion was supported by molecular dynamics simulations, and the probable mechanism responsible for synergism was discussed. In addition, an analysis allowing accurate determination of the concentration regimes, where the synergistic effect can be expected, was given. It was shown that for the two-phase system, the *n*-octanol molecule preadsorption at the liquid/gas interface causes an increase in C*_n_*TAB adsorption coverage over the level expected from its equilibrium value in the one-component solution. In the case of the three-phase system, the synergistic effect was related to the ionic surfactants serving as an anchor layer for *n*-octanol, which, in water/*n*-octanol solution (one-component system), do not adsorb on the surface of quartz.

## 1 Introduction

Multiphase systems with interfaces of complex (mixed) adsorption layers are ubiquitous in nature, as well as in numerous technical applications. Important examples of such systems are foams, emulsions, paints, surfactant solutions, complicated microemulsions, foamed emulsions, double emulsions, biological cells, liposomes, etc.^1,2^ The properties of such complex multicomponent systems are largely determined by the dynamic properties of the interfacial layers, which can be composed of surfactants, polymers, biopolymers, nanoparticles, and their mixtures. The increased interest in multiphase systems is based on their importance for various applications in pharmaceuticals, cosmetics, food production, biotechnology, biomedicine, and mineral processing.^3−7^ The dynamic behavior of such systems is complex because it depends on the composition, structure, and various internal relaxation processes within the interfacial layers, which in turn strongly depend on the dynamics of the contacting fluid phases.

One of the important factors determining the above-mentioned interface properties is the composition of adsorption layers. In the case of a multicomponent interface, the interactions between mixed adsorption layer molecules can result in better macroscopic system properties than expected from each component separately. This enhancement of the considered effects is called synergism. In the literature, it is well known that binary surfactant mixtures are more effective in modification of such parameters as surface tension, foamability, or floatability at lower concentrations than pure surfactant solutions. For example, Yoon and Ravishankar^8^ showed a synergistic effect of cationic/nonionic (dodecylamine hydrochloride/octanol) surfactant solutions on flotation of silica. Addition of a nonionic surfactant increased significantly the recovery of silica particles; i.e., at a dodecylamine concentration of 1 × 10^–5^ M, the recovery was equal to ca. 20%, and for the mixture, it was ca. 90%. Mixed surfactant systems are also used in an enhanced oil recovery technique to decrease the interfacial tension in oil–water systems more than individual surfactants and get better recovery of trapped oil from natural oil reservoirs, for example, nonyl-phenol-ethoxylated-carboxylate/quaternary ammonium chloride as an anionic/cationic mixture^9^ and sodium dodecyl sulfate/cetyltrimethylammonium bromide also as an anionic–cationic mixture.^10^ Jiang et al.^11^ showed the synergistic effect on foamability between sodium dodecyl sulfate as an anionic hydrocarbon surfactant and an amphoteric short-chain fluorocarbon surfactant (Capstone, FS-50) with six carbon atoms. Addition of FS-50 to the solution of anionic surfactant increased the initial foam height; i.e., at a sodium dodecyl sulfate concentration of 1 × 10^–3^ M, the foam height was ca. 40 mm, and after addition 0.1 wt % FS-50 to the mixture, the height increased to ca. 130 mm. Despite the fact that the synergistic effect has been observed for various systems, reports attempting for elucidation of the origin of synergism as well as surfactant concentration ranges, where this effect can be expected, are very scarce. It can be deduced that the synergism should be a consequence of interactions between the mixture constituents, either in the bulk or on the surface (inside the mixed adsorption layer). Therefore, to explain the mechanism of synergistic effects, the intermolecular interactions between surfactants have to be considered.

The paper shows the results of systematic studies on the synergistic effect of a model two-component system, i.e., a mixture of a cationic quaternary amine of different carbon chain lengths and simple nonionic alcohol. It is shown that the existence of the synergistic effect depends on the mutual relation between mixture component concentrations, adsorption kinetics, and consequently their adsorption coverages at the interfaces, determining the magnitude of interactions between adsorbed species in the mixed adsorption layer. It is shown that the synergistic effect can be observed in both two-phase and three-phase systems.

## 2 Materials and Methods

### 2.1 Materials

Alkyltrimethylammonium bromides (C*_n_*TAB, where *n* is the number of carbon atoms in the alkyl chain), i.e., octyltrimethylammonium bromide (*n* = 8), dodecyltrimethylammonium bromide (*n* = 12), cetyltrimethylammonium bromide (*n* = 16), and octadecyltrimethylammonium bromide (*n* = 18) of highest purity (≥98%, Sigma-Aldrich), were used as cationic surfactants, while *n*-octanol (≥98%, VWR) was used as a nonionic surfactant.

Particles of high-purity quartz (>98% SiO_2_) of size 50–100 μm were used in floatability tests.

Milli-Q water (18.2 MΩ·cm) was used for cleaning all parts of the experimental setup and preparation of pure solutions of cationic surfactants and their mixtures with a nonionic surface-active substance used in the experiments.

The experiment was designed in such a way that the concentration of C*_n_*TAB, as the solution main component, was changed in a quite broad range, while the concentration of *n*-octanol (nonionic additive) was kept constant and equal to 5 × 10^–4^ M. This particular concentration was chosen as the lowest, having a significant and easily measurable synergistic influence on the measured parameters.^12^

### 2.2 Foamability

Foamability and foam stability of pure C*_n_*TAB solutions of various concentrations and their mixtures with *n*-octanol were assessed using a Dynamic Foam Analyzer (DFA100, KRÜSS GmbH) apparatus. The apparatus consisted of (i) a cylindrical column with prisms, (ii) two parallel electrodes with seven sensors to measure, based on conductivity, the evolution of the foam liquid content, and (iii) two vertical rows of photodiodes as light sources (blue - λ = 469 mm) and light scanners for simultaneous automatic measurement of foam (*H*_f_) and solution (*H*_s_) heights as a function of time. At the bottom of the column, the filter paper (as an air disperser), made of chemically pure cellulose, of the pore size equal to 12–15 μm, was sealed. Prior to each experiment, the column was carefully washed in diluted Mucasol (a commercially available cleaning liquid, purchased from Sigma-Aldrich), and then rinsed with a large amount of Milli-Q water. After connection of the filter paper with the column at the DFA stand, the column was filled with 50 mL of the studied solution. The air was pumped through the air disperser at a flow rate of 0.5 L/min for 20 s, and the *H*_f_, *H*_s_, and liquid content were measured and recorded by PC, employing ADVANCE software (KRÜSS GmbH). The experiments were carried out at room temperature (22 ± 1 °C) and natural pH of liquid solutions (ca. 6).

### 2.3 Floatability

Flotation tests were carried out in an XFLB laboratory flotation machine with an automatic scrapper and a cell of volume 0.75 dm^3^. The quartz particles (weight ca. 50 g) were added to the flotation cell previously filled with 0.75 dm^3^ of the studied solution and then conditioned for 60 s at a rotor speed of 1950 rpm without air introduction. After 60 s of conditioning, the air was introduced to the system with a constant airflow rate equal to 0.2 m^3^/h. Each fraction of quartz floated as a function of time was automatically collected by a scrapper. Floating and nonfloating fractions were dried at 80 °C and then weighed to calculate the flotation recovery. The experiments were carried out at room temperature (22 °C ± 1°) and natural pH of liquid solutions (ca. 6).

### 2.4 Dynamic and Equilibrium Surface Tension Measurements

To analyze the influence of the mixed adsorption layers on the performance of quite dynamic multiphase systems (foam column, flotation cell), the values of dynamic surface tension were used. The values of dynamic surface tension of one-component and mixed solutions of C*_n_*TAB and 5 × 10^–4^ M *n*-octanol were determined according to the maximum bubble pressure (MBP) method using a BPA-1S maximum bubble pressure tensiometer with a capillary diameter of 0.13 mm (SINTERFACE). In this method, the pressure is measured as a function of the flow rate of air, and the surface tension is calculated from the measured maximum bubble pressure using the Laplace equation.

Equilibrium values of surface tension in one-component solutions of C*_n_*TAB of various concentration and 5 × 10^–4^ M *n*-octanol were determined via the pendant drop profile analysis technique using a KRÜSS DSA100 apparatus.

### 2.5 Molecular Dynamics Simulations

The Gromacs 2019.2 package^13,14^ with the CHARMM^15^ force field was used for all-atom molecular dynamics (MD) modeling. The system setup and parameters were adapted from Yazhgur et al.^16^ Briefly, for the cetyltrimethylammonium cation (C_16_TAB), the CHARMM36-saturated lipid model was used.^17^ For *n*-octanol, the compatible CHARMM general force field was used.^18^ Bromide ion parameters were taken from Horinek et al.^19^ For water, the modified TIP3P model of CHARMM was applied.^15,20^ The structure, topology, and parameters for the (001) quartz surface were adapted from the INTERFACE force field^21−24^ and generated using Nanomaterial Modeler in the CHARMM-GUI web server.^25−27^ The degree of ionization for quartz was set to 8.6%, which corresponds to pH 5.6.^22^

All MD simulations were run at constant temperature and volume (NVT ensemble) conditions. Temperature coupling was controlled via a V-rescale thermostat^28^ at temperature 298 K and coupling constant 0.5 ps. van der Waals interactions were described by the Lennard–Jones potential, smoothly shifted to zero between 1.0 and 1.2 nm. The electrostatic interactions were modeled by the PME method,^29^ corrected for the slab geometry,^30^ with a 1.2 nm cutoff, 0.12 nm grid spacing, and fourth-order splines. The equations of motion were integrated using the leap-frog integration scheme and a 2 fs time step. Bonds involving hydrogen were constrained using LINCS^31^ and SETTLE^32^ algorithms. All molecular visualizations employ the VMD software package.^33^

The radial distribution function and deuterium order parameter *S*_CD_ were calculated using built-in GROMACS tools. The *S*_CD_ was calculated using formula
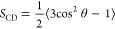
1where θ is the angle between the C–H bond and a vector normal to the liquid/gas interface. The angular brackets denote a time and ensemble average. For the CH_2_ chains oriented normal to the interface, the *S*_CD_ value approaches −0.5. For chains lying on the interface and rotating freely around their long axis, *S*_CD_ approaches 0.25.

For the simulations at the liquid/gas interface, the system was a periodic rectangular simulation box, 8 × 8 × 24 nm^3^, consisting of a slab of water of thickness ∼8 nm, separated by a vacuum region. Initial configurations, generated using PACKMOL,^34^ were constructed by randomly placing 34 C_16_TAB molecules, and additional 394 *n*-octanol molecules in the case of a C_16_TAB/*n*-octanol mixture, into two surfactant monolayers at opposite orientations. Surfactant headgroups were oriented toward the water slab, while the exact angle between the tail and the interface was chosen randomly. The number of surfactants corresponds to their surface concentrations Γ_Octanol_ = 5.1 × 10^–6^ [mol/m^2^] and Γ_C_16_TAB_ = 4.5 × 10^–7^ [mol/m^2^]. The surface concentrations were set to match the values determined experimentally for the separate *n*-octanol and C_16_TAB solutions at bulk concentrations *c*_*n*-octanol_ = 5 × 10^–4^ [mol/dm^3^] and *c*_C_16_TAB_ = 1 × 10^–5^ [mol/dm^3^]. The C_16_TAB and *n*-octanol molecular structures and the initial configurations are presented in Figure S1 (see the Supporting Information).

For the solid/liquid interface, the ≈3 nm thick quartz slab was separated by a water region, and the simulation box size was 8.35 × 8.5 × 20 nm^3^. The 32 C_16_TAB molecules, with headgroups oriented toward the quartz/water interface, were placed on both sides of quartz, similar to in the case of the simulations at the liquid/gas interface. The 32 *n*-octanol molecules, however, were randomly placed in the bulk solution. The initial configurations are shown in Figure S2 (see the Supporting Information).

To make the simulation systems charge-neutral, an adequate number of Br^–^ ions were added. After 200 steps of energy minimization, the systems were simulated for 70 ns. Based on the previous simulations of similar systems,^16^ the first 20 ns were considered as the initial equilibration period and disregarded from the analysis. For the simulations at the solid/liquid interface, the diffusion of octanol molecules in the bulk solution needed to be considered. As this could influence the equilibration time, the production runs for these systems were extended by the next 70 ns. However, no significant changes in the systems were observed for the additional simulation.

## 3 Results and Discussion

### 3.1 Foamability

**Figure 1:**
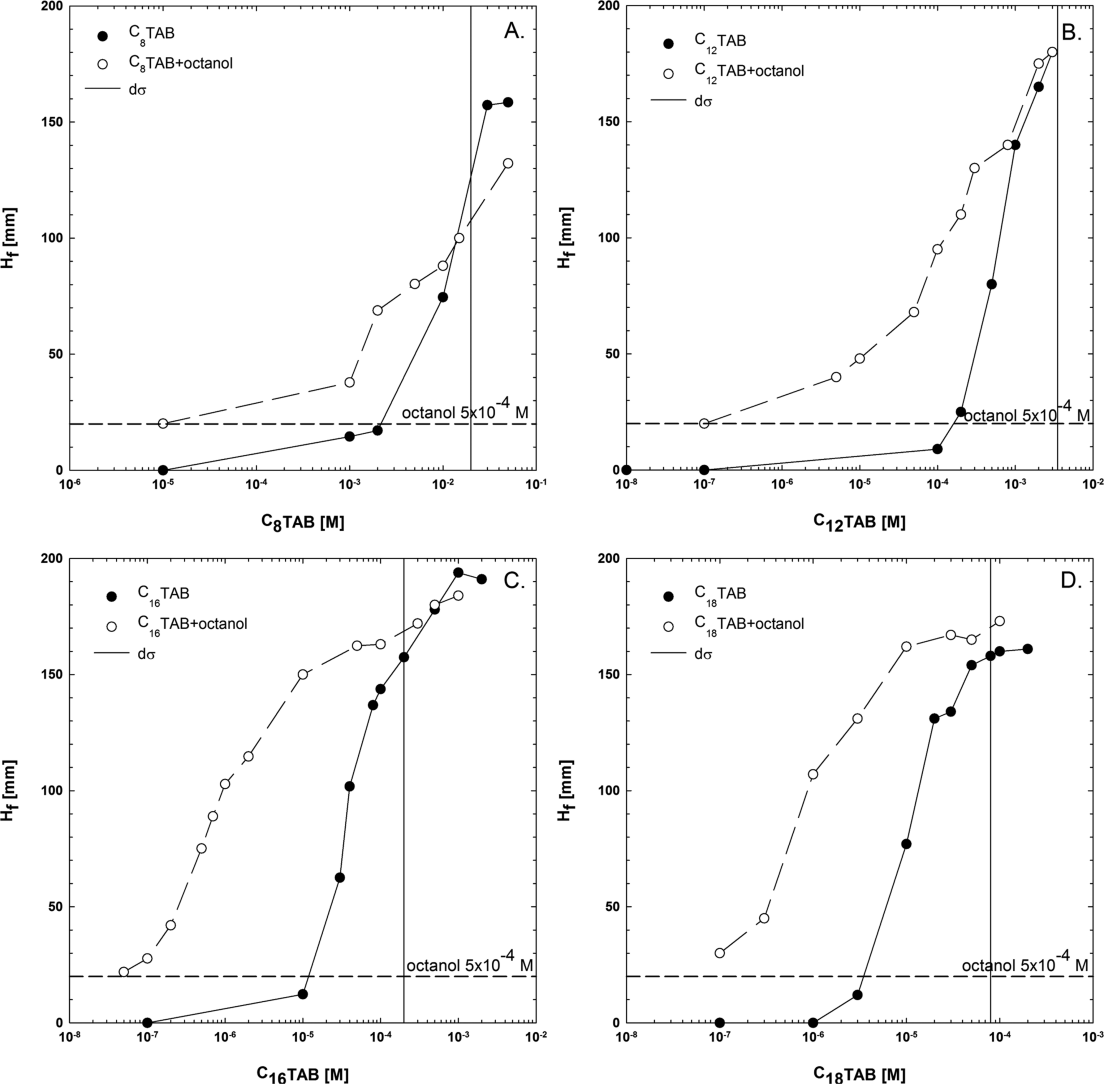
Height of foam after the foaming time is equal to 20 s for (A) C_8_TAB, (B) C_12_TAB, (C) C_16_TAB, and (D) C_18_TAB solutions of various concentrations and their mixtures with 5 × 10^–4^ M *n*-octanol. The height of foam for pure 5 × 10^–4^ M *n*-octanol solution is marked in the figure as a horizontal dashed line (CSCs are marked by vertical solid lines).

Foam heights (*H*_f_) for pure C*_n_*TAB (with *n* equal to 8, 12, 16, and 18) solutions and their mixtures with the *n*-octanol solution of concentration 5 × 10^–4^ M, for foaming time *t*_f_ = 20 s, are presented in Figure 1. The vertical line represents the critical synergistic concentration (CSC)^12,35^ determined from the equation
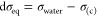
2where σ_water_ and σ_(c)_ are the equilibrium surface tensions of water and surfactant solution of a given concentration, respectively. The CSC in the case of solution foamability performance was the characteristic concentration of C*_n_*TAB in a mixture with *n*-octanol, above which the synergistic effect, i.e., significant enhancement of foamability of the C*_n_*TAB solution by the nonionic surfactant addition, was no longer visible. A detailed explanation of the approach used for the CSC determination from the surface tension isotherms of corresponding surface-active substances, as well as the importance of the dσ_eq_ parameter and discussion on synergistic effect existence for solution foamability, has been given in our previous papers.^12,35^ Briefly, the foamability CSC value of a cationic/nonionic surfactant mixture can be determined on the basis of dσ_eq_ values (eq 2) of the main solution component (cationic surfactant) determined from the surface tension isotherm. Such an approach has a certain physical meaning—dσ_eq_ is proportional to the surface dilatational modulus *E*, which is a parameter of crucial role in the stability of foam films in wet (transient) foams under dynamic conditions, and can be associated with different, concentration-dependent participations of nonionic additives in the mixed adsorption layer at the solution/air (bubble) interface.^12,35^ In the present study, different approaches for analysis of the synergistic effect were proposed. In the following (Section 4), we present the elaborated protocol.

As seen in Figure 1, the presented trend of *H*_f_ variations with the solution concentration is similar for all studied substances. For example, in the case of C_8_TAB, for a pure solution of concentration equal to 1 × 10^–5^ M, no foam was observed. In the presence of 5 × 10^–4^ M *n*-octanol, as the nonionic additive, the *H*_*f*_ was higher and equal to ca. 20 mm. This was also the *H*_f_ value characteristic for pure 5 × 10^–4^ M *n*-octanol solution. A similar effect can be observed also for higher C_8_TAB concentrations—the presence of the nonionic surfactant increased the solution foamability. However, above the CSC, in this case, equal to ca. 2 × 10^–2^ M, this trend starts to be opposite—the *H*_f_ was comparable for pure C_8_TAB and mixed C_8_TAB/*n*-octanol solutions and the synergistic effect disappeared. Similar trends can be observed for other studied C*_n_*TAB solutions, and foamability results, for pure and mixed solutions, are presented in Figure 1B–D. The CSCs determined on the basis of the dσ_eq_ values, marked with vertical lines, for C_12_TAB, C_16_TAB, and C_18_TAB were equal to 3 × 10^–3^, 2 × 10^–4^, and 1 × 10^–4^ M, respectively. It is worth mentioning here that the most significant synergistic effect was observed for the C_16_TAB and C_18_TAB solutions of concentrations equal to 1 × 10^–5^ and 1 × 10^–6^ M, respectively. The highest differences between the *H*_f_ values determined for solutions with and without *n*-octanol addition were determined there. The weakest synergistic effect, compared to the other C*_n_*TAB solutions, was observed for C_8_TAB practically in all concentration ranges.

### 3.2 Floatability of Quartz

**Figure 2:**
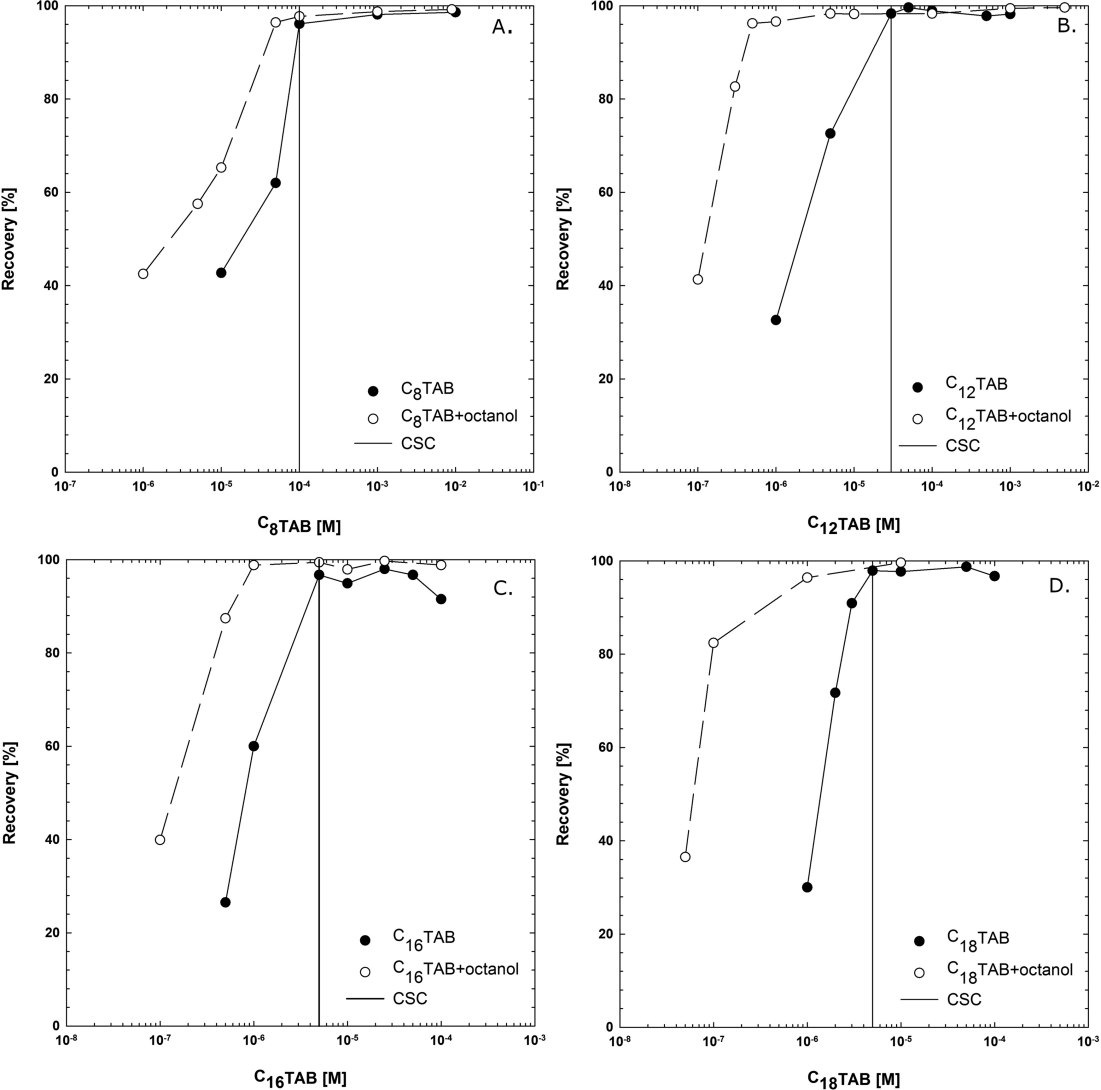
Recovery of quartz for (A) C_8_TAB, (B) C_12_TAB, (C) C_16_TAB, and (D) C_18_TAB solutions of various concentrations and their mixtures with 5 × 10^–4^ M *n*-octanol (CSCs are marked by vertical solid lines).

For the three-phase system, the synergistic effects in C*_n_*TAB/*n*-octanol mixtures were studied on the basis of flotation experiments conducted using the laboratory flotation machine. The results of quartz flotation in C*_n_*TAB solutions of various concentrations and their mixtures with 5 × 10^–4^ M *n*-octanol are presented in Figure 2, where data on the flotation recovery as a function of the concentration of the main solution component (cationic surfactant) are shown. It is seen that the recovery of quartz increased with increasing concentrations of C*_n_*TAB; however, the characteristic concentration range of the main solution component, in the presence of *n*-octanol, as the nonionic additive, significantly shifted toward smaller concentration values. It is worth mentioning that the recovery of quartz in pure water was equal to 0% and the recovery either less or equal to ca. 40% for small concentrations of the studied C*_n_*TAB solutions might have been attributed to the mechanical entrainment of quartz in the flotation machine.

Figure 2A presents the results of flotation experiments for C_8_TAB. As seen in Figure 2A, addition of 5 × 10^–4^ M *n*-octanol to C_8_TAB solutions slightly increased the quartz recovery; e.g., for the concentrations equal to 1 × 10^–5^ and 5 × 10^–5^ M, the quartz recovery was equal to ca. 40 and 60%, while in the mixtures, the quartz recovery was equal to ca. 65 and 95%, respectively. The value of critical synergistic concentration (CSC) from flotation experiments was determined as an intersection between the dependence for pure and mixed solutions (no synergistic effect), and for C_8_TAB, the CSC was equal to 1 × 10^–4^ M.

Corresponding results for C_12_TAB are shown in Figure 2B. As seen in this case, the presence of *n*-octanol caused a much stronger synergistic effect, visible even for low concentrations of the cationic surfactant. For example, at the C_12_TAB concentration of 1 × 10^–6^ M, the recovery was equal to ca. 30% and increased remarkably for the mixture to ca. 95%, i.e., was more than three times. The CSC value determined for C_12_TAB from flotation tests was equal to 3 × 10^–5^ M. For C_16_TAB and C_18_TAB, the above-discussed trends, presented in Figure 2C,D, respectively, are similar. The magnitude of the synergistic effect is comparable to the C_12_TAB case. The values of CSC determined from flotation tests were equal to 5 × 10^–6^ M for both C_16_TAB and C_18_TAB.

The obtained results revealed that the presence of the nonionic surfactant additive in the cationic surfactant solution changed the concentration regimes, where specific flotation recovery of quartz particles can be achieved. As seen in Figure 2, a similar flotation response could be obtained for the pure and mixed systems, for which the concentration of the main solution component (cationic surfactant) differed more than an order of magnitude. The strongest synergistic effect for quartz flotation in mixed solutions was revealed for relatively low C*_n_*TAB concentrations.

### 3.3 Molecular Dynamics Simulations

**Figure 3:**
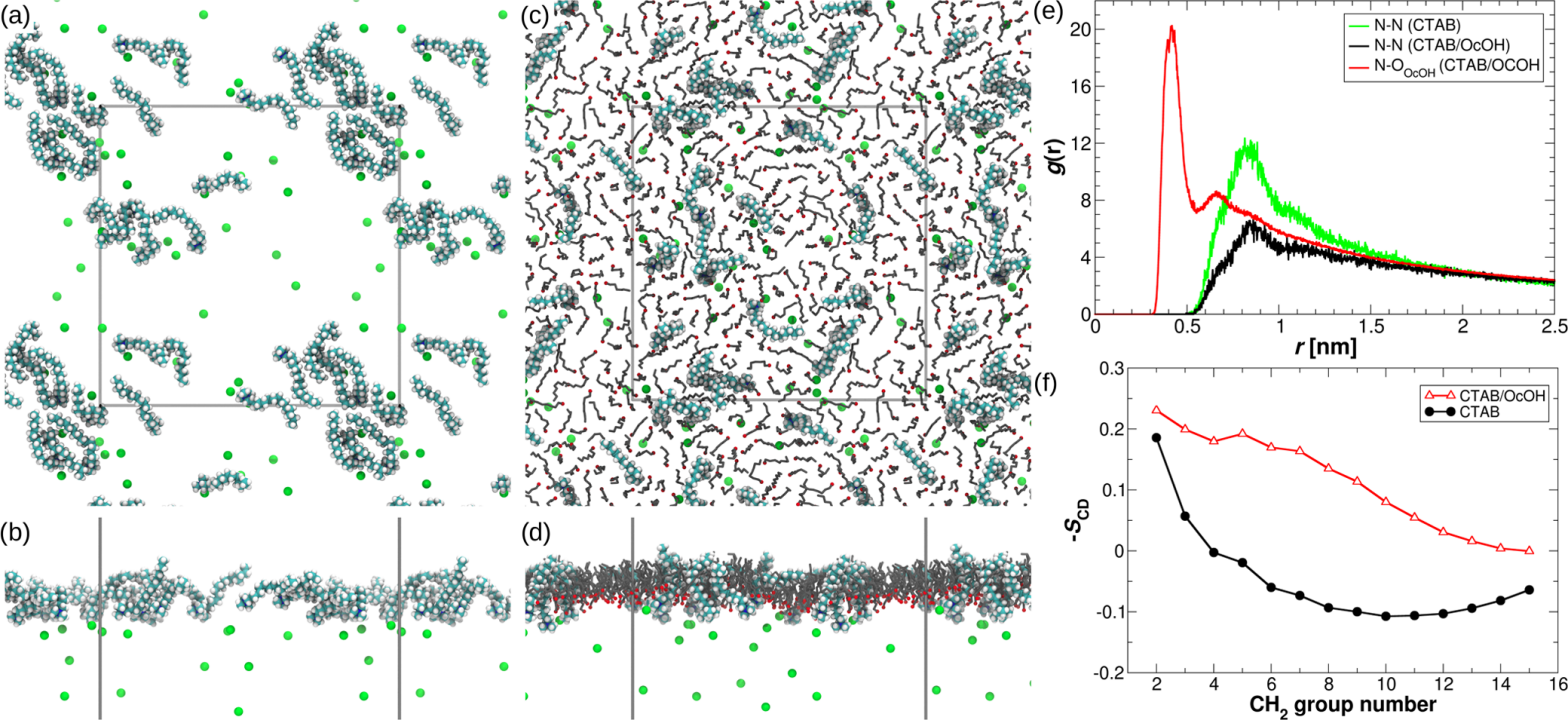
Snapshots of the MD configurations after 70 ns production run corresponding to the system with C_16_TAB-only (a, b) and C_16_TAB/octanol mixture (c, d), where (b) and (d) show the side views. Br^–^ ions are presented as green spheres. Gray lines represent the periodic boundaries of the simulation box. Water was omitted for clarity. (e) Radial distribution function between the C_16_TAB headgroups, as well as between the C_16_TAB headgroup and OH group of *n*-octanol. (f) Deuterium order parameter, S_CD_, for the CTAB tails as a function of the CH_2_ group number, starting from the CTAB headgroup.

The experimental results of foamability and floatability measurements in one-component and mixed solutions revealed that, despite the simplicity of the mixture system applied, there exists a synergistic effect, causing a significant enhancement of the observed experimental parameters. To analyze and understand the molecular origin of this phenomenon, the MD simulations of the corresponding systems were performed. The MD simulations proved to be a valuable tool capable of explaining the experimental observation in C*_n_*TAB-covered interfaces via changes in the interaction and organization.^16^ Here, the experimental data clearly indicates the synergistic effect of mixed ionic and nonionic surfactants. The systems containing C_16_TAB and the C_16_TAB/*n*-octanol mixture were simulated at the liquid/gas interface. The left side of Figure 3 shows the surfactant organization at the interface, after 70 ns production run, of one-component and mixed solutions. The amounts of *n*-octanol and C_16_TAB correspond to the surface concentration determined from the experimental data and reflect the bulk concentrations equal to 5 × 10^–4^ and 1×10^–5^ M, respectively. To gain additional insight into the structure and organization of the surfactant molecules, the radial distribution function (RDF) and order parameter *S*_CD_ (eq 1) were calculated and are presented in Figure 3e,f, respectively. The changes in the C_16_TAB ordering, induced by the presence of *n*-octanol, are pronounced and can be quantitatively investigated via *S*_CD_. As recently shown,^16^ an increase in the C_16_TAB surface concentration results in a shift toward tail orientation normal to the interface, i.e., the C_16_TAB molecules are more ordered. Here, the presence of *n*-octanol induced changes in the C_16_TAB orientation similar to the increase in the C_16_TAB surface concentration by an order of magnitude.^16^

The different structural organization of C_16_TAB is visible in Figure 3a,c. In the case of the pure C_16_TAB solution, the C_16_TAB clusters are formed, while in the C_16_TAB/*n*-octanol mixture, the C_16_TAB seems to be more dispersed. To investigate this observation quantitatively, the RDF between the C_16_TAB headgroups and between the C_16_TAB headgroup and OH group of *n*-octanol were calculated and are shown in Figure 3e. Indeed, the presence of *n*-octanol molecules significantly decreases the RDF between the C_16_TAB headgroups, making C_16_TAB molecules more separated from each other. Additionally, the high peak of *g*(*r*) between C_16_TAB and *n*-octanol, at a distance close to 0.5 nm, suggests that C_16_TAB is mainly surrounded by the *n*-octanol groups. This can be explained by strong electrostatic repulsions between the C_16_TAB headgroups. In the one-component system, however, the electrostatic repulsions seem to be suppressed by the attractive interaction between the hydrophobic tails. Additionally, C_16_TAB in the one-component system is more immersed in water compared to the C_16_TAB/octanol system. The mean numbers of water molecules within 0.5 nm from the single C_16_TAB molecule for pure C_16_TAB and CTAB/*n*-octanol systems are 107 ± 5 and 88 ± 3, respectively.

The mobility of the C_16_TAB molecules in the monolayer was studied via the mean-squared displacement (MSD) in the *z* direction (normal to the interface) and the lateral diffusion coefficient *D* in the *xy* plane. As can be expected, the lateral diffusion of C_16_TAB molecules within the monolayer containing *n*-octanol is lower due to the steric and volume excluded effects (see Figure S3a in the Supporting Information). The C_16_TAB headgroup diffusion coefficients *D* for the one-component and C_16_TAB/*n*-octanol systems are equal to 2.9 ± 0.7 × 10^–5^ and 0.55 ± 0.25 × 10^–5^ [cm^2^/s], respectively. Interestingly, the MSD in the *z* direction of the C_16_TAB headgroup in the C_16_TAB/*n*-octanol mixture (see Figure S3b in the Supporting Information) is lower by about 20% in comparison with that in the pure C_16_TAB solution. The attractive interactions between the C_16_TAB and octanol hydrophobic tails stabilize the C_16_TAB in the monolayer. The mobility of the C_16_TAB headgroups in the *z* direction might be associated with the C_16_TAB ability to move into the bulk solution; i.e., the transfer of C_16_TAB molecules from the interface to the bulk solution is hindered in the presence of *n*-octanol. As the experimental system is in dynamic equilibrium, the addition of *n*-octanol is expected to slow down the diffusion of C_16_TAB from the interface to the bulk solution, i.e., shifting the equilibrium constant toward a higher concentration of C_16_TAB at the interface.

**Figure 4:**
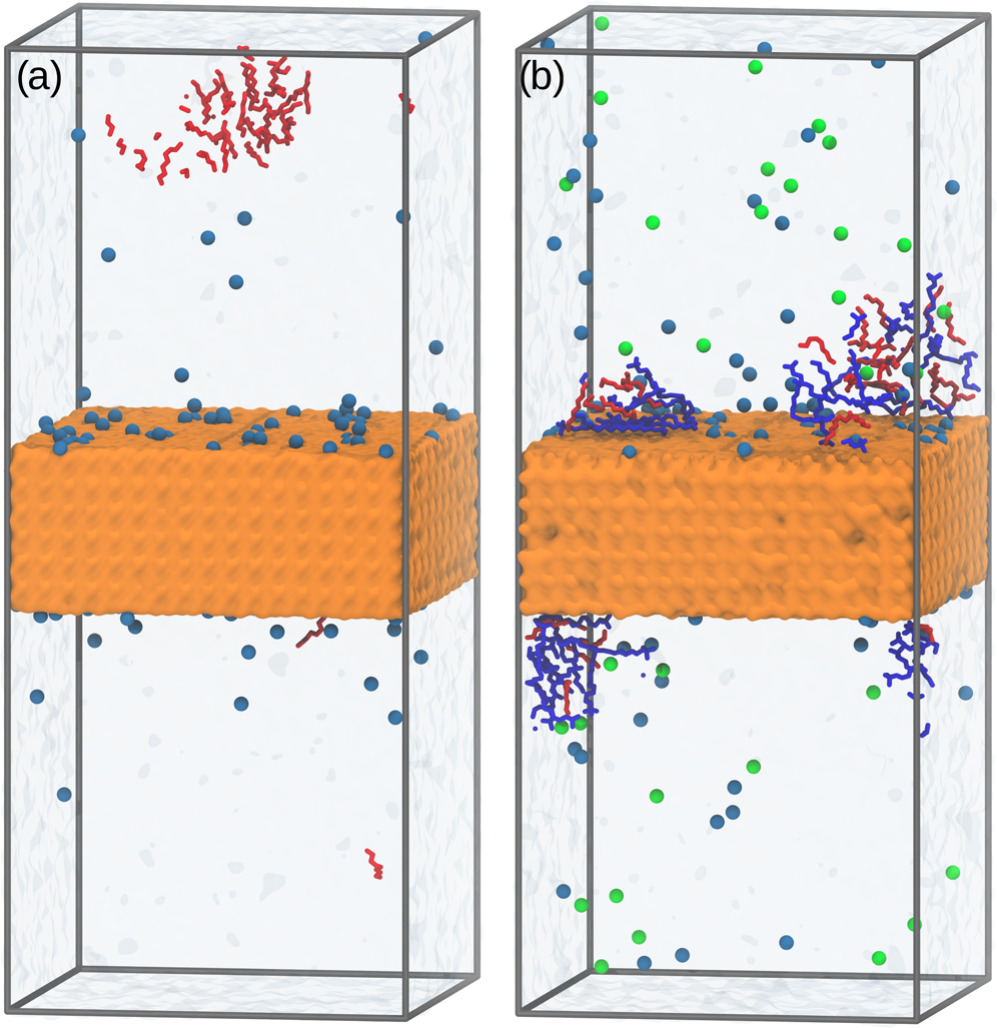
Snapshots of the MD configurations after 70 ns production run corresponding to the system with *n*-octanol solution (a) and (b) *n*-octanol/C_16_TAB mixture at (001) quartz/water interface. Br^–^ and Na^+^ ions are presented as green and light blue spheres. Gray lines represent the periodic boundaries of the simulations box. For clarity, water is presented using surface representation and hydrogen atoms of C_16_TAB (blue) and *n*-octanol (red) molecules are omitted.

To interpret the corresponding experimental results in the three-phase system, the MD simulations of the *n*-octanol solution and *n*-octanol/C_16_TAB mixture at the quartz/water interface were also performed. As the MD simulations of bulk solution at extremely low surfactant concentrations are not efficient due to the large simulation box, significantly higher concentrations were considered. Therefore, the obtained results provide rather qualitative information. Nevertheless, the findings from the MD simulations show the crucial role of C_16_TAB in *n*-octanol adsorption on the quartz surface. As seen in Figure 4, in the case of quartz immersed in the *n*-octanol solution, almost no adsorption onto the solid surface was observed. This is in line with the literature reports, showing no change in the surface wettability compared to the pure water solution,^36,37^ and agrees with experimental data obtained in this study—there was no quartz particle flotation in the pure *n*-octanol solution.

Instead, as shown in Figure 4a, the *n*-octanol molecules form droplets in water, which is related to the concentration of *n*-octanol exceeding the solubility limit. After the addition of C_16_TAB, however, the *n*-octanol molecules are present on the quartz surface. The positively charged C_16_TAB headgroups adsorb on the quartz surface via electrostatic interactions. Then, the C_16_TAB hydrophobic tails exposed to water act as an anchor layer for the *n*-octanol hydrophobic tails. Due to the relatively low degree of ionization of OH groups on quartz, the adsorbed C_16_TAB molecules are separated from each other and do not form the monolayer even at higher concentrations. It is expected that the organization of C_16_TAB at the quartz/water interface is dictated by the quartz surface charge density, i.e., solution pH; at a relatively high surface charge, the monolayer of C_16_TAB may be formed.

## 4 Analysis of the Synergistic Effect Mechanism

### 4.1 Two-Phase System (Liquid/Gas Interface)

To analyze the mechanism of the experimentally observed synergistic effect and determine accurately its concentration regimes, the data on dynamic surface tension, σ(*t*), were used. The analysis of the σ(*t*) values was based on a simple assumption, being simultaneously the definition of synergism, that some characteristic effect observed in the mixture of C*_n_*TAB/*n*-octanol is higher than that expected from individual constituents. In our case, this effect was, as mentioned above, the foamability enhancement (modification of liquid/gas interface properties).

**Figure 5:**
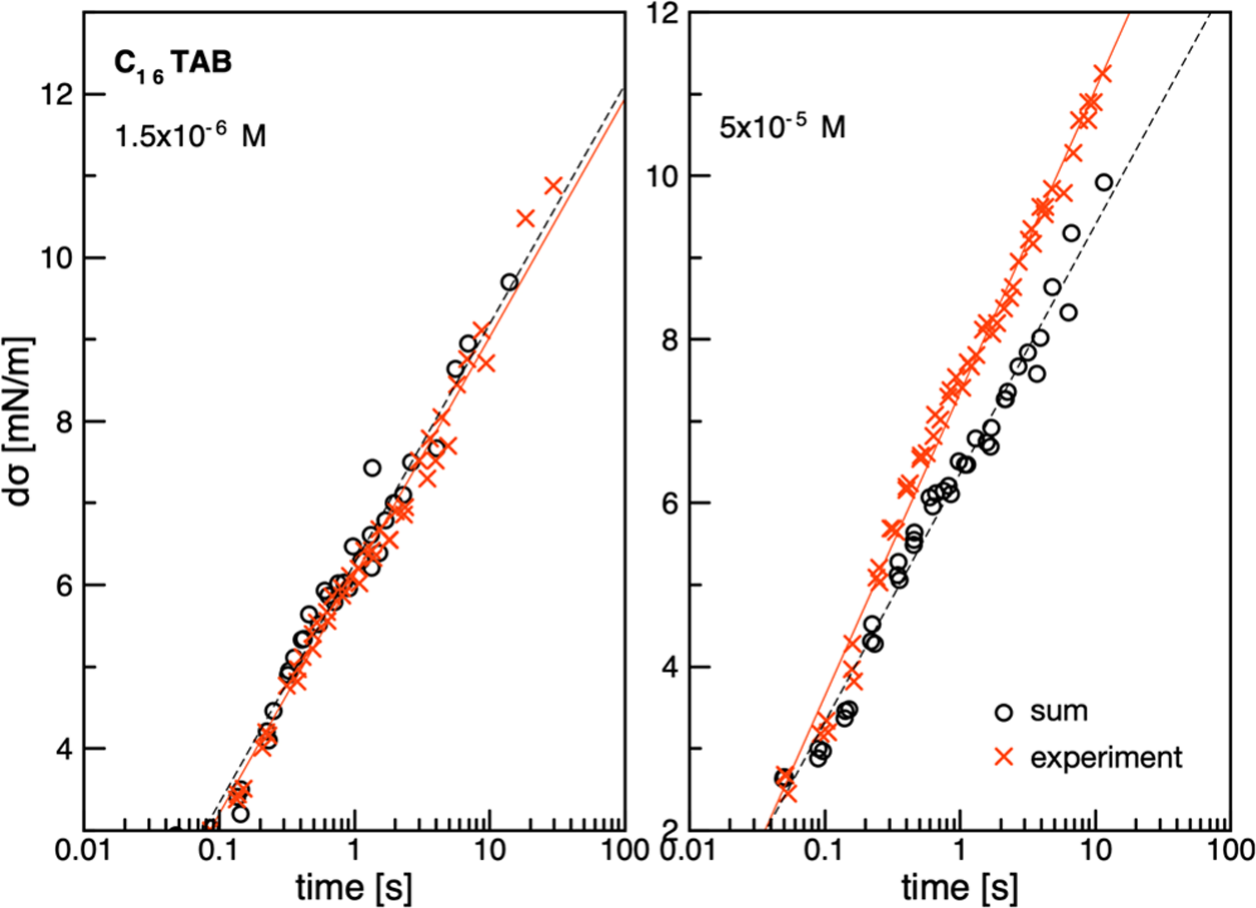
Values of dσ_exp_(*t*) and dσ_sum_(*t*) (calculated according to eqs 3–7) as a function of time with fitted linear regression lines for determination of their line*a*r slopes (parameters *a*) for two chosen C_16_TAB concentrations. The *a* parameters were used for assessment of the degree of surface tension decrease in mixed C*_n_*TAB solutions, according to the protocol described in the text.

**Figure 6:**
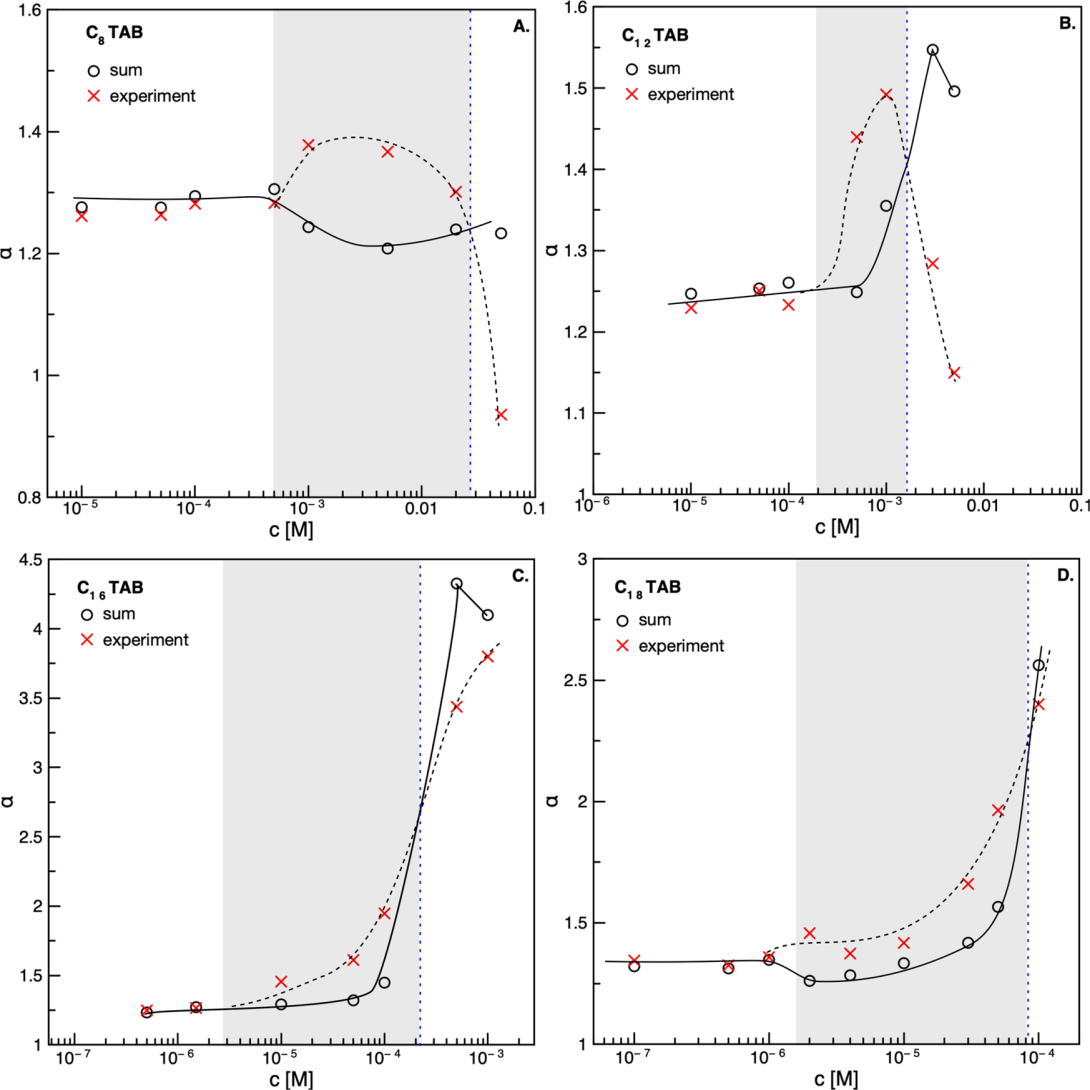
Linear slopes (parameters *a*_sum_ and *a*_exp_) determined for all mixed solutions of studied C*_n_*TAB with 5 × 10^–4^ M *n*-octanol, according to the protocol presented in Figure 5 and described in the related text. (A): *n* = 8, (B) *n* = 12, (C) *n* = 16, (D) *n* = 18. Solid and dashed lines added to guide the eye.

To visualize directly the synergism existence, the following analysis protocol was proposed. Taking the dynamic surface tension data, the values of dσ_exp_(*t*) were calculated as
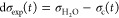
3where σ_c_(*t*) represents the dynamic surface tension values determined for the mixed solution, where subscript *c* corresponds to the concentration of C*_n_*TAB, while σ_H_2_O_ is the water surface tension. Then, hypothetical dσ_sum_(*t*) values were calculated as
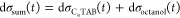
4where
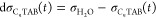
5
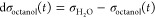
6assuming that the ability of the surface tension decrease in the mixture is a simple sum of the characteristic effects from each of the mixture’s components, either pure C*_n_*TAB solutions of given concentrations (σ_C*_n_*TAB_(*t*)) or *n*-octanol of concentration equal to 5 × 10^–4^ M (σ_octanol_(*t*)). The dσ_sum_(*t*) values were calculated for the corresponding similar time ranges. To perform the analysis, calculated dσ_exp_(*t*) and dσ_sum_(*t*) values for chosen C*_n_*TAB concentrations were plotted as a function of time. Examples of such plots are presented in Figure 5 for two randomly chosen C_16_TAB concentrations. To check whether the synergistic effect really exists and to assess its magnitude, the linear regression in the form
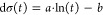
7was fitted to the dσ(*t*) (dσ_exp_(*t*) and dσ_sum_(*t*)) data, and the slope coefficient, *a*, was calculated. A comparison of the value of this coefficient for dσ_exp_(*t*) and dσ_sum_(*t*) dependences allows for direct assessment of the synergistic effect magnitude. If the value of *a* calculated for dσ_exp_(*t*) was higher than that determined for dσ_sum_(*t*), i.e., when *a*_sum_ < *a*_exp_, the synergistic effect existed because the experimentally observed decrease in surface tension with time was higher than that predicted from the adsorption performance of the individual mixture components. Otherwise, when *a*_sum_ ≥ *a*_exp_, the synergistic effect was negligible. The calculated coefficients for the studied C*_n_*TAB solutions of the chosen concentrations are presented in Figure 6. As seen, the synergistic effect (*a*_sum_ < *a*_exp_) existed only in some specific concentration ranges, which agrees with the results of foamability experiments. Moreover, the presented analysis allowed determining very accurately the concentration regimes where the synergistic effect could be expected, as well as the values of critical synergistic concentration (CSC). The determined CSC values were in very good agreement with those determined from the foamability experiments and the values reported in our previous studies.^12,35^

The analysis presented in Figures 5 and 6 resulted in three main conclusions. For mixtures, where the concentration of C*_n_*TAB was low and where, consequently, the *a*_sum_ ≈ *a*_exp_, the adsorption of the mixture components was comparable and corresponded to the characteristic adsorption kinetics of each of the surface-active substances. Due to faster adsorption and difference in the concentration (*n*-octanol was used in excess here), the *n*-octanol molecules were the main constituents of the mixed adsorption layer. When the concentration of the C*_n_*TAB in the mixture increased, *a*_sum_ became larger than *a*_exp_, which indicated the synergistic effect existence. Close and above the CSC, where *a*_sum_ ≥ *a*_exp_, the synergistic effect disappeared, most probably due to the competitive adsorption of the mixture components at the liquid/gas interface (of comparable bulk concentrations).

### 4.2 Three-Phase System (Liquid/Gas and Liquid/Solid Interfaces)

**Figure 7:**
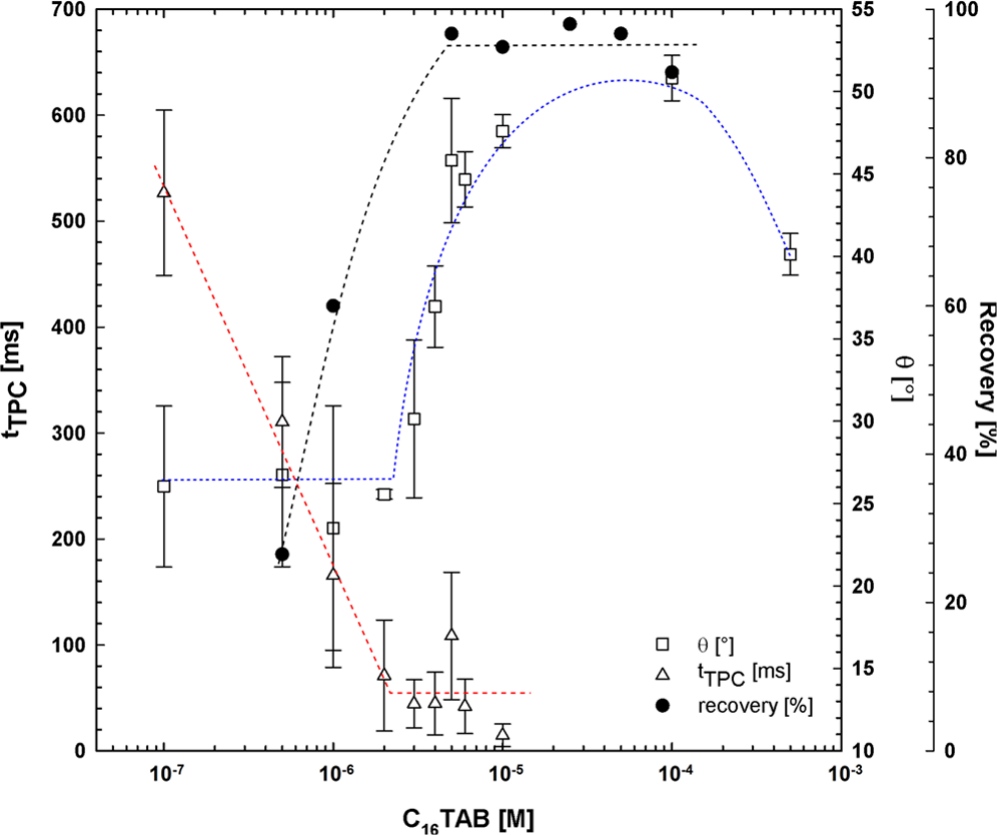
Time of three-phase contact formation (t_TPC_), advancing contact angle (θ), and flotation recovery of quartz as a function of C_16_TAB concentration. Data on *t*_TPC_ and θ were taken from ref (37).

To elucidate the origin of synergism between the cationic/nonionic surfactants in quartz flotation, first, factors affecting the quartz recovery in pure C*_n_*TAB solutions have to be analyzed. This analysis is done here for C_16_TAB, as an example. The results of our study and available literature data show that for C_16_TAB solutions of different concentrations, there is a clear correlation between the quartz surface zeta potential, advancing contact angle (θ) (sessile drop), time of three-phase contact (*t*_TPC_) formation (single bubble measurements), and flotation recovery.^37,38^ In Figure 7, the results for the flotation recovery (shown in Figure 2 for pure C_16_TAB) are set together with the data on *t*_TPC_ and θ. The point of zero charge of quartz surface in C_16_TAB is equal to ca. 5 × 10^–5^ M.^38^ This concentration value is in the range of 1 × 10^–5^–1 × 10^–4^, where the maximum value of the contact angle was determined.^36−39^ The existence of the maximum contact angle value (θ_max_) means that in the range of corresponding concentration there is a monolayer of C_16_TAB molecules on the quartz surface. As seen in Figure 7, around the values of θ_max_, also the flotation recovery is at maximum (almost 100%), while the *t*_TPC_ value, after a rather steep decrease, starts to be constant. Such θ and *t*_TPC_ behavior vs concentration was attributed to two different mechanisms of rupture of the intervening liquid (wetting) film formed during the bubble collision with the solution/quartz interface.^37,40−43^ More detailed analysis of these mechanisms is out of the scope of this paper. Here, we would like to underline that the θ and *t*_TPC_ values strongly correlate with the flotation response. Figure 7, however, shows the results for pure C_16_TAB solutions of different concentrations. Does the same correlation hold also for mixed solutions with nonionic surfactant addition? Below, we focus on the origin of the synergistic effect related to the *n*-octanol presence, as shown in Figure 2.

To explain the synergistic effect for quartz flotation observed in C*_n_*TAB/*n*-octanol solutions, the role of the nonionic component of the mixtures has to be elucidated. Three phases in contact with the two-component solution of mixed surfactants constitute a complex system. Therefore, the direct correlation between *t*_TPC_, θ, and flotation recovery is much more difficult compared to the one-component solution (Figure 7). According to the literature, the addition of *n*-octanol to C_16_TAB solutions alters the kinetics of the three-phase contact formation by single bubble collision with the quartz surface. Yoon and Ravishankar^8^ showed that the hydrophobicity of the mica (aluminum silicate) surface increased in the presence of *n*-octanol in dodecylamine solution and this effect was attributed to *n*-octanol molecule coadsorption between hydrocarbon chains of the cationic surfactant, replacing water molecules. As a consequence, the C_16_TAB molecules can be much more closely packed. The results of our MD simulations confirm these observations.

On the other hand, it was shown^36,37^ that the *n*-octanol molecules in the C_16_TAB solutions adsorb mainly at the gas/liquid interface, contributing to a decrease in mixed solution surface tension and a decrease in the rising bubble velocity as well as the time of bubble attachment to the solid surface. The positively charged cationic surfactant interacts much stronger with the negatively charged quartz surface than nonionic *n*-octanol molecules. This mechanism is consistent with the results of foamability experiments and is described elsewhere.^12,35^ The synergistic effect on the mixed solution foamability performance can be attributed to different, concentration-dependent contributions of the nonionic surfactant into the mixed adsorption layer at the liquid/gas interface. Despite the fact that the contribution of *n*-octanol in the mixed adsorption layer is different and depends on the concentration, its presence significantly reduces the rising bubble velocity.^37^ Smaller rising velocity can increase the flotation recovery by increasing the bubble–quartz particle collision probability and efficiency of attachment (three-phase contact formation) by prolongation of the contact time. Certainly, as shown by MD simulations, in the case of the three-phase system, the synergistic effect can be distributed (divided) between the liquid/gas and liquid/solid interfaces. The ratio between the magnitude of the effect characteristic for either the fluid or solid interface should depend on the surfactant type and properties of the solid surface.

**Figure 8:**
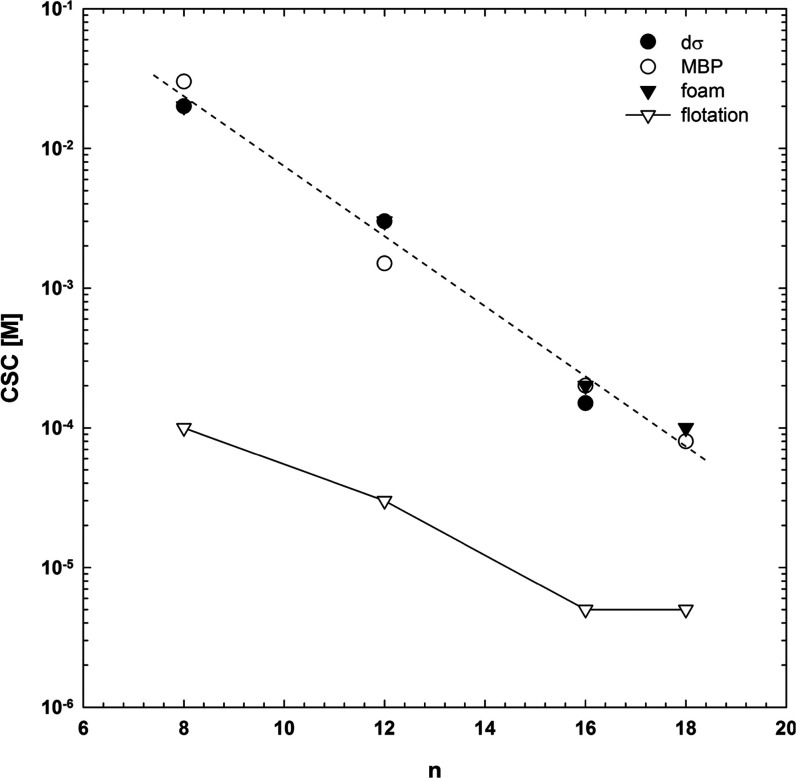
Effect of the number of carbon atoms in the alkyl chain (*n*) of C*_n_*TAB on the critical synergistic concentration (CSC) determined from the maximum bubble pressure (Figure 6), foamability (eq 2 and Figure 1), and floatability (Figure 2).

Figure 8 presents the dependence of the CSC values determined from foamability and floatability experiments for C*_n_*TAB solutions with different numbers of carbons in the alkyl chain (n). In the case of foamability, the values of CSC were taken directly from Figure 1 (intersection between dependence for pure and mixed solutions) and from the dσ_eq_ calculations (eq 2). The CSC values for floatability of quartz were determined on the basis of Figure 2. As seen, there is a perfect agreement between values of the CSC determined for foamability according to the two above-mentioned approaches, i.e., from foam height experiments and dσ calculations (either equilibrium or dynamic surface tension values). Such a good agreement means that the CSC for mixed solutions can be very easily predicted by the data on the surface tension of pure components, without the need for performing foamability experiments. Moreover, as can be observed in Figure 8, the CSC values determined from flotation experiments are much lower compared to foamability tests (where only the liquid/gas interface was involved). Nevertheless, the trend is identical to an almost constant difference in the C*_n_*TAB concentration for both presented curves (determined from floatability and foamability) equal to about two orders of magnitude. This can be considered as direct proof for the additive character of the synergistic effect, which for the three-phase system is caused by the synergism of mixed adsorption layers formed both at the liquid/gas and liquid/solid interfaces.

## 5 Conclusions

The synergistic effects in binary surfactant mixtures on foamability (two-phase system) and floatability of quartz (three-phase system) were investigated using four cationic alkyltrimethylammonium bromides (C*_n_*TAB, with *n* = 8, 12, 16, 18) and *n*-octanol as the nonionic surfactant. It was found that the addition of *n*-octanol increased the foamability of C*_n_*TAB solutions and floatability of quartz beyond the limit expected from simple additive effects. The synergistic effect of *n*-octanol could be observed for all of the studied C*_n_*TAB below the threshold concentration, called the critical synergistic concentration (CSC). Above this concentration, the positive effect of the *n*-octanol presence was either negligible or started to reduce the foamability of pure C*_n_*TAB solutions—the antagonistic effect. To analyze and understand the molecular origin of this phenomenon, a detailed analysis was performed, which was supported by the molecular dynamics simulations for two-phase and three-phase systems. This allows determining very accurately the concentration regimes where the synergistic effect can be expected in both two-phase and three-phase systems.

**Figure 9:**
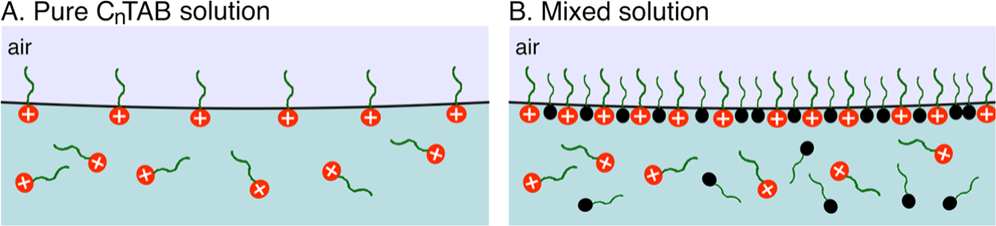
Schematic illustration of the synergistic effect origin in the two-phase system–adsorption layer at the liquid/gas interface in (A) pure C*_n_*TAB solution and (B) mixed solution of C*_n_*TAB and *n*-octanol. Due to *n*-octanol presence, the adsorption coverage of C*_n_*TAB molecules can be much higher compared to that expected from its equilibrium value in the one-component solution.

**Figure 10:**
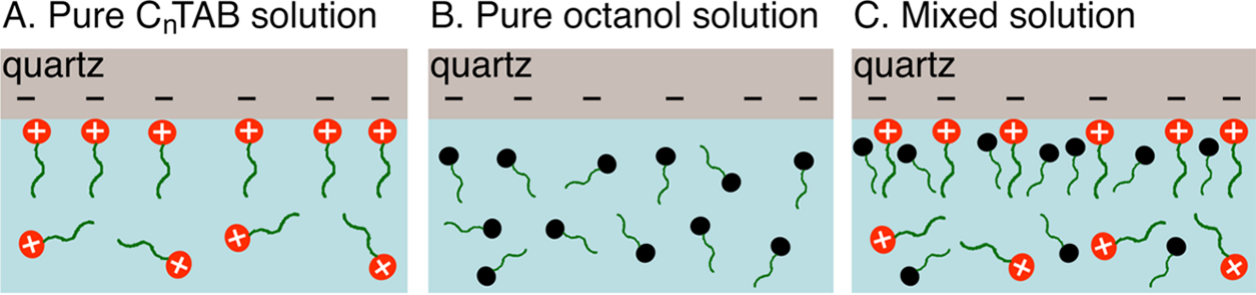
Schematic illustration of the synergistic effect origin in the three-phase system: (A) adsorption layer of C*_n_*TAB molecules at the quartz (solid/liquid) interface, (B) no adsorption of the nonionic surfactant on the solid surface immersed in the one-component *n*-octanol solution, and (C) ionic surfactants serving as an anchor layer for *n*-octanol—the ability of *n*-octanol to incorporate into the mixed adsorption layer leads to an increase in the solid surface hydrophobicity above the level characteristic for C*_n_*TAB alone.

It was shown that for blend solutions, in the specific C*_n_*TAB concentration ranges, a remarkable increase in the solution foamability and floatability of quartz particles can be achieved. This increase could not be explained by a simple increase of the total surfactant concentration, as the overall effect was higher than expected from the adsorption behavior of each of the individual components of the mixture at the liquid/gas and liquid/solid interfaces. To elucidate the mechanism of synergism determined in the experiments, the MD simulations were employed. The main findings are presented in Figures 9 and 10, where schematic illustrations of the origin of the observed synergistic effect in two-phase and three-phase systems are given.

For the two-phase system, the addition of *n*-octanol to the C*_n_*TAB solution resulted in an increase of the C*_n_*TAB molecule surface concentration (Γ) at the liquid/gas interface (see Figure 9B). This increase was much higher compared to that expected from the equilibrium Γ value in the one-component solution (Figure 9B). As shown by the MD simulations, this effect should be mainly related to slower diffusion of C*_n_*TAB molecules from the interface to the bulk solution, i.e., the effect of the shifting of the equilibrium constant toward higher concentrations of C*_n_*TAB at the interface, in the presence of *n*-octanol.

In the three-phase system, the mechanism of synergism was different due to the fact that *n*-octanol itself, in contrast to the cationic surfactant, could not adsorb on the quartz surface (compare the illustrations in Figure 10A,B). In this case, the synergistic effect was related to the ionic surfactants serving as an anchor layer for *n*-octanol (Figure 10C), which, in a one-component solution, do not adsorb on the quartz surface.

## Supporting Information Available

Initial configurations for MD simulations, surfactant molecular structures, sodium ion distribution on quartz, and mean-squared displacement of CTAB molecules (PDF)
