# Supplementary material for: Synergistic Effect of Binary Surfactant Mixtures in Two-Phase and Three-Phase Systems
Source: J Phys Chem B. 2021 Apr 13;125(15):3855–66. doi: 10.1021/acs.jpcb.1c00664 (PMC8154601; doi:10.1021/acs.jpcb.1c00664)
Supplement: Supplementary file 1 — jp1c00664_si_001.pdf [file jp1c00664_si_001.pdf]

Supporting Information for

Synergistic Effect of Binary Surfactant Mixtures in Two-phase and Three-phase Systems

Agata Wiertel-Pochopien [1*], Piotr Batys [1], Jan Zawala [1*], Przemyslaw B. Kowalczuk [2,3]

[1] *Jerzy Haber Institute of Catalysis and Surface Chemistry, Polish Academy of Sciences, Niezapominajek 8, 30-239 Krakow, Poland*

[2] *Norwegian University of Science and Technology, Department of Geoscience and Petroleum, S. P. Andersens veg 15a, 7031 Trondheim*

[3] *Wroclaw University of Science and Technology, Faculty of Chemistry, Wybrzeze Wyspianskiego 27, 50-370 Wroclaw, Poland*

[*] Corresponding authors: jan.zawala@ikifp.edu.pl; agata.wiertel-pochopien@ikifp.edu.pl

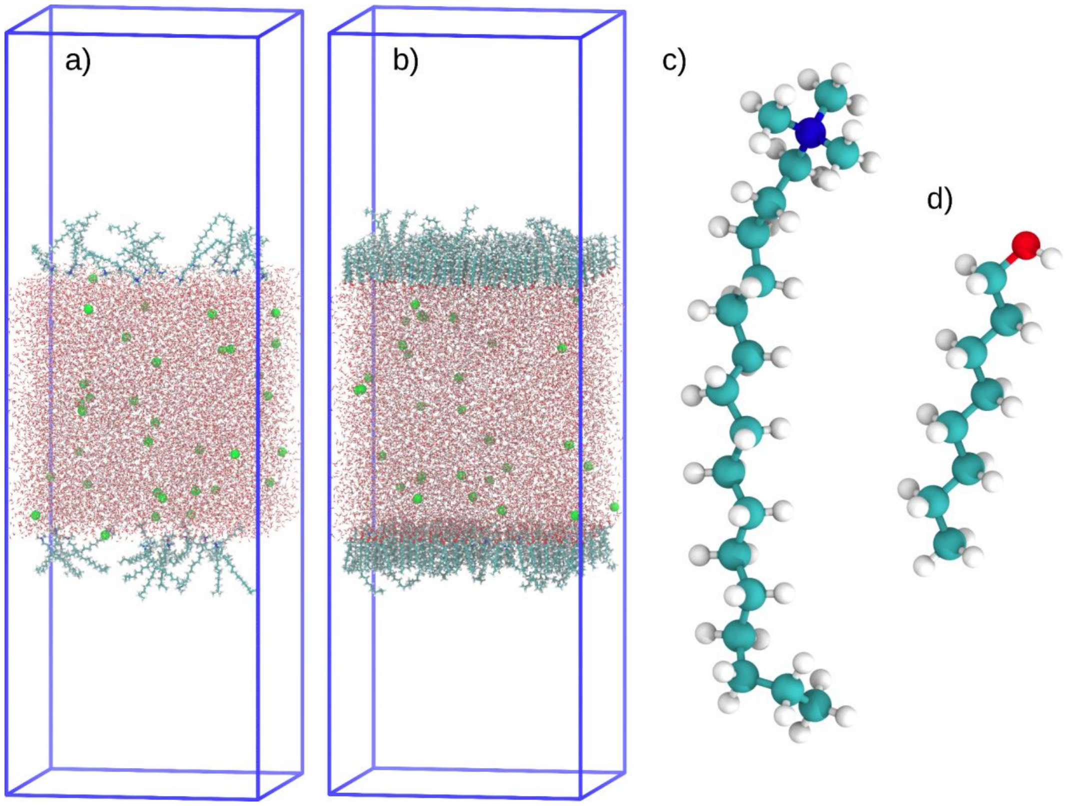

Figure S1. Snapshots showing initial configurations of CTAB only (a) and CTAB/OcOH mixture (b), as well as the molecular structure of CTAB (c) and OcOH (d).

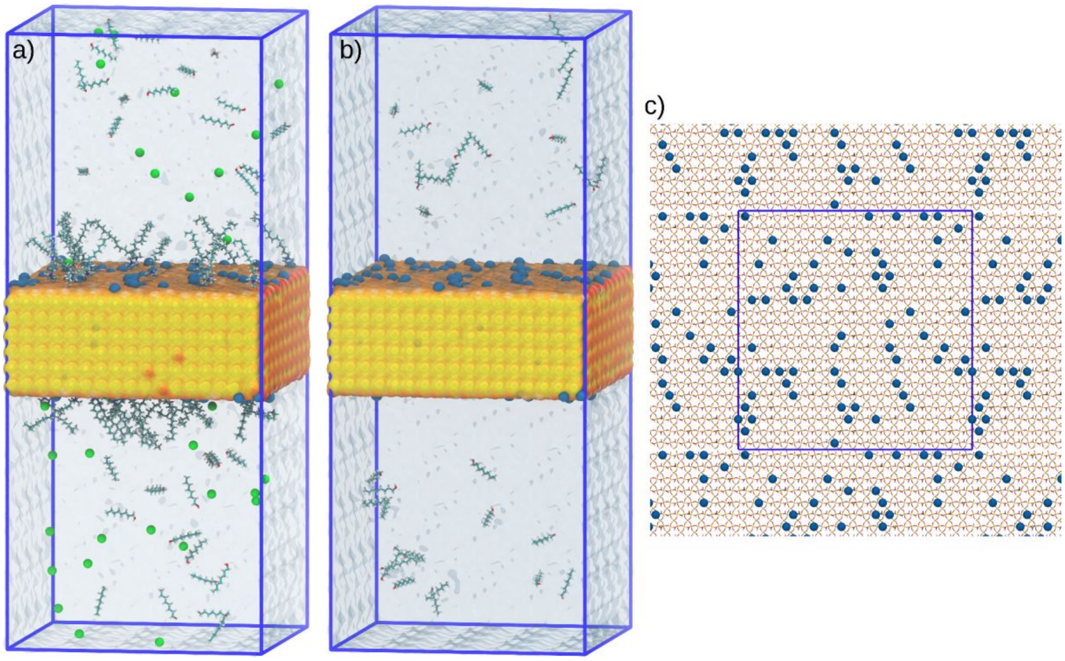

Figure S2. Snapshots showing initial configurations of OcOH solution (a) and OcOH/CTAB mixture (b) at (001) quartz/water interface. (c) The initial distribution of Na^+ ions on (001) quartz. For the clarity, water is presented using surface representation.

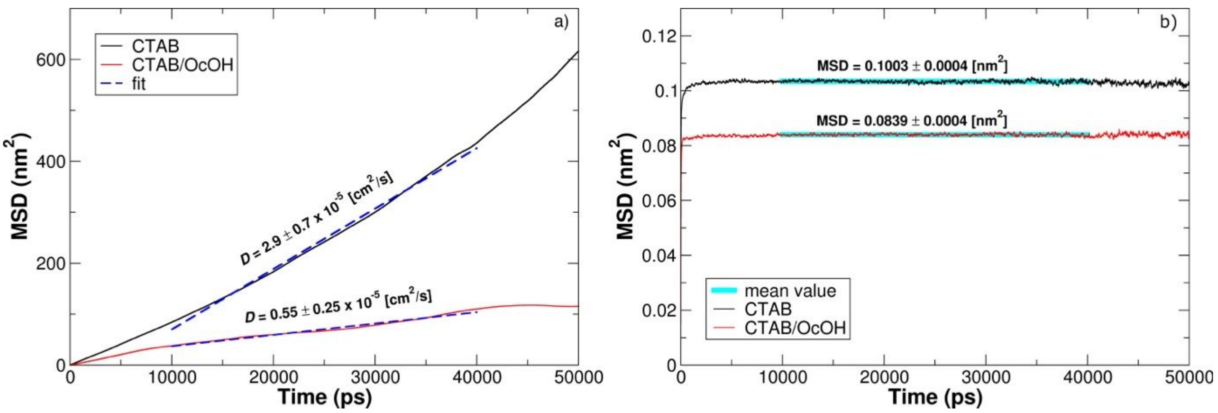

Figure S3. Mean squared displacement (MSD) in the *xy* plane (a) and the *z* direction (b). The diffusion coefficients *D* were determined using the Einstein relation, within the range from 10000 to 40000 [ps]. To exclude the effect of the whole system fluctuation in *xyz* directions, the center of mass motion was removed.
